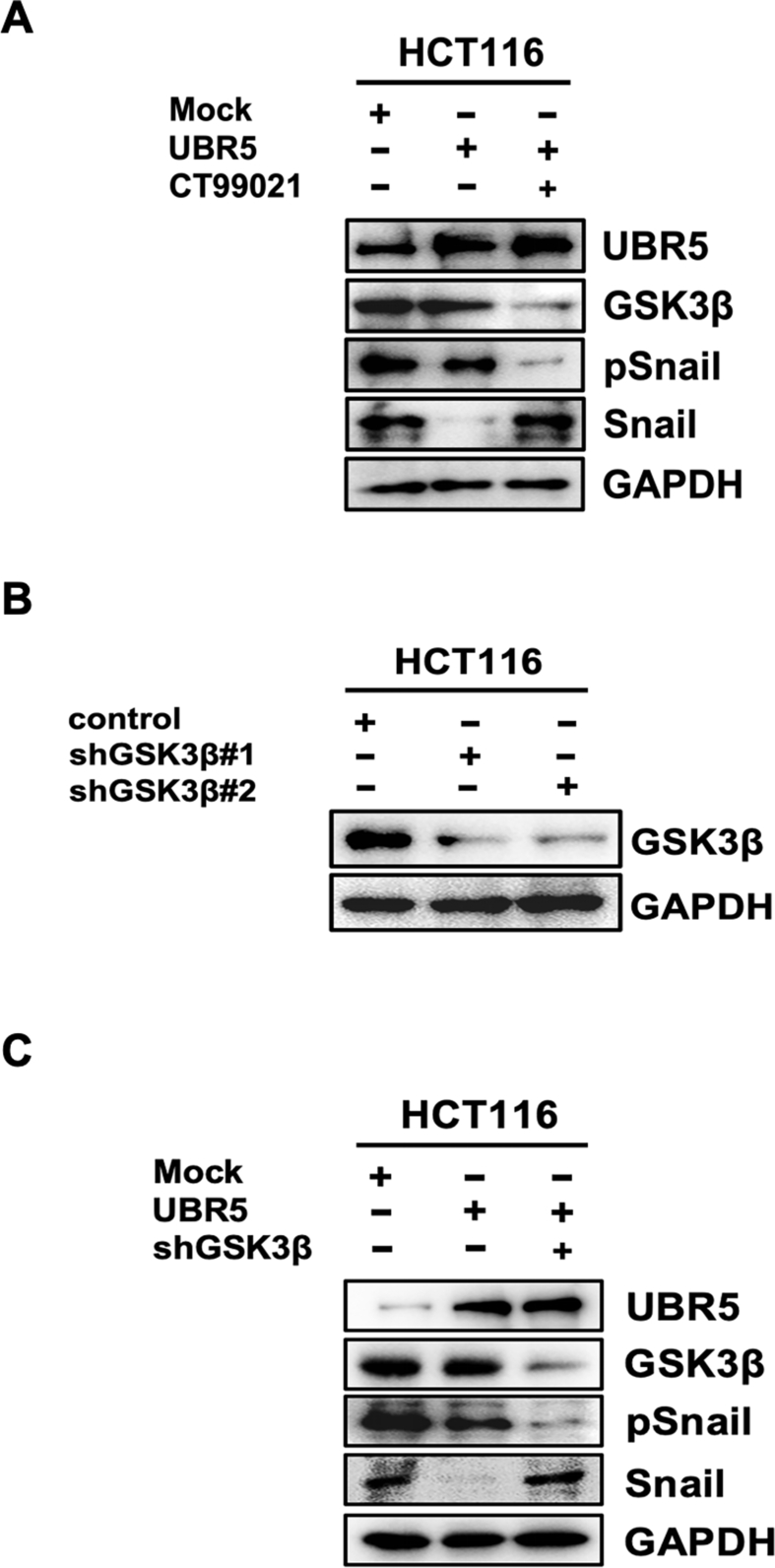# UBR5 regulates the progression of colorectal cancer cells through Snail-induced epithelial–mesenchymal transition

**DOI:** 10.1016/j.gendis.2025.101679

**Published:** 2025-05-13

**Authors:** Xinyue Zhao, Ruiying Liu, Zhihui Han, Zehao Li, Ling Mei, Yuyang Liu, Xueqi Fu, Yue Jin

**Affiliations:** a Edmond H. Fischer Signal Transduction Laboratory, School of Life Sciences, Jilin University, Changchun, Jilin 130012, China; b National Engineering Laboratory of AIDS Vaccine, School of Life Sciences, Jilin University, Changchun, Jilin 130012, China; c Key Laboratory of Organ Regeneration & Transplantation of the Ministry of Education, Jilin University, Changchun, Jilin 130061, China

**Keywords:** Colorectal cancer, EMT, Metastasis, Snail, UBR5

## Abstract

Snail is a core inducer of epithelial-to-mesenchymal transition. Here, we show that UBR5 promotes ubiquitin-mediated degradation of Snail and regulates the progression of colorectal cancer cells through its E3 ubiquitin ligase function. UBR5 specifically binds to Snail *in vitro*, but not Slug, and its degradation depends on snail phosphorylation. Depletion of endogenous UBR5 causes Snail protein accumulation, epithelial-to-mesenchymal transition, and tumor invasion in colorectal cancer cells. Conversely, the overexpression of UBR5 reduces Snail protein abundance and cellular invasiveness. The activity-deficient mutant UBR5 C2768S disrupts its binding and degradation to Snail, thereby losing the ability to regulate epithelial-to-mesenchymal transition in colorectal cancer cells. UBR5 is lowly expressed in human colorectal cancer versus normal tissues, and high UBR5 levels correlate with favorable prognosis, suggesting that UBR5 sustains the epithelial state and inhibits cancer progression. These findings establish the UBR5-Snail axis as a mechanism of post-translational regulation of epithelial-to-mesenchymal transition and colorectal cancer metastasis.

## Introduction

Ranked as the third most common cancer globally, colorectal cancer (CRC) poses a considerable challenge to public health worldwide.^1^ Although advances in CRC screening and therapeutic strategies have substantially reduced mortality, approximately 20%–30% of patients are still diagnosed with metastatic disease, resulting in a dismal 5-year survival rate of less than 15%.^2^^,^^3^ CRC, often considered a cancer of older adults, is increasingly affecting younger populations, with cases rising by 1.5% annually among young adults.^4^ Thus, unraveling the genetic alterations that drive the initiation and progression of CRC, along with the molecular mechanisms that enable metastasis, has become a critical focus in the quest for targeted therapies.

Cancer progression involves events from simple cell expansion to complex distant colonization. Tumor metastasis involves successive, interconnected steps.^5^^,^^6^ Epithelial-mesenchymal transition (EMT) plays a critical role in cancer metastasis, where epithelial cells adopt mesenchymal traits, leading to enhanced cell motility and invasive potential. This process involves the loss of cell-cell junctions and polarity, allowing non-motile, polarized epithelial cells to transition into highly motile, invasive mesenchymal cells.^7^ E-cadherin down-regulation plays a crucial role in driving EMT, making it a defining marker of epithelial cell identity and its loss a hallmark of the transition toward a mesenchymal state.^8^ Research has also shown that EMT promotes metastasis in CRC. KRAS mutations and TGF-β signaling activation enhance the metastatic potential of CRC cells by promoting EMT, intravasation/extravasation, and colonization of secondary organs.^9^

Snail1, a zinc finger transcription factor, was first identified in *Drosophila* as a repressor of shotgun (an E-cadherin homologue) transcription. It regulates large-scale cell movements during mesoderm formation and neural crest delamination.^10^^,^^11^ The Snail family of transcription factors trigger the EMT program in development, fibrosis, and cancer.^12^ These proteins interact with E-box-containing DNA promoters, facilitating cancer cell invasiveness and metastasis in several malignancies.^13^, ^14^, ^15^, ^16^ Within the large Snail-centered EMT network, E3 ligases, such as FBXW1,^17^ FBXL5,^18^ FBXO11,^19^ and TRIM21,^20^ interact with Snail and regulate tumor cell invasion metastasis and proliferation.

Ubiquitin protein ligase E3 component N-recognin 5 (UBR5), also termed EDD1, is an E3 ligase containing the ubiquitin binding domain,^20^ the ubiquitin-protein ligase E3 component N-recognize domain,^21^ and a catalytic C-terminus domain (HECT).^22^ UBR5, a highly conserved protein in metazoans, plays a key role in regulating the DNA damage response, cell cycle, metabolism, transcription,^23^, ^24^, ^25^ and apoptosis. UBR5 plays diverse roles in cancer, functioning either as a tumor suppressor or an oncogene, depending on its E3 ubiquitinase activity and the specific substrates it targets. For instance, UBR5 facilitates tumor growth in gallbladder cancer by activating the PI3K/AKT signaling pathway and contributes to CRC progression by targeting and degrading the tumor suppressor protein esophageal cancer-related gene 4 (ECRG4).^26^ UBR5 overexpression could promote the degradation of capping actin protein of muscle Z-line subunit alpha 1 (CAPZA1), preventing metastasis in pancreatic cancer.^27^ Oncogene-like functions of UBR5 in regulating ovarian cancer-tumor microenvironment crosstalk have also been noted.^28^

Although substantial progress has been made in understanding EMT and CRC metastasis, the precise molecular mechanisms underlying these processes are still not fully elucidated. The role of the large HECT E3 subfamily in regulating Snail protein homeostasis has been explored in only a few studies. The precise roles of various molecules or signaling pathways in CRC metastasis through EMT warrant further investigation. Additionally, effective strategies targeting these critical molecules or pathways to inhibit EMT and prevent CRC metastasis are yet to be developed. In this study, we hypothesized that the E3 ubiquitin ligase UBR5 regulated the stability of Snail protein via the proteasomal degradation system, thereby influencing the EMT process in CRC metastasis. By investigating the role of UBR5 and its catalytic inactivation mutations in Snail degradation and cancer cell invasion, this research aimed to provide new strategies and targets for CRC treatment.

## Materials and methods

### Cell culture and transfection

The cell lines involved in this study are preserved in the Fisher Laboratory, College of Life Sciences, Jilin University. HEK293T and SW480 cells were cultured in Dulbecco's modified Eagle's medium (Gibco, Waltham, USA, C11995500BT) supplemented with 10% fetal bovine serum (Kangyuan Biotechnology, Shanghai, China, KY-01000). HCT116 and SW620 cells were cultured in RPMI-1640 medium (Gibco, C11875500BT) supplemented with 10% fetal bovine serum. All cells were cultured in an incubator at 37 °C with 5% CO_2_. Transfection was performed using Lipofectamine 2000 (Invitrogen, Carlsbad, CA, USA) according to the manufacturer's instructions.

### Plasmids and mutagenesis

The UBR5, truncated and mutated UBR5, and Snail and Snail 6SA mutated cDNAs were synthesized and cloned into the lentiviral vector pLVX-IRES-neo by Miaoling Biology (Wuhan, China). The UBR5 truncated and mutated cDNA was cloned into the pLVX-IRES-neo vector with a Myc tag. The Snail and Snail with mutated 6SA were cloned into the pLVX-IRES-neo vector with a Flag tag. Snail full-length and different truncated mutants of Snail and UBR5 were cloned into the pET28a vector.

### Western blotting and immunoprecipitation assays

Western blotting was performed as previously described.^29^ Whole cell extracts were prepared using cold RIPA lysate buffer (150 mM NaCl, 50 mM Tris PH 7.5, 1% NP-40, 10% Glycerol). Proteins were separated on 6%–12% gels, depending on molecular weight, and transferred to a PVDF membrane (Millipore, Shanghai, China, IPVH00010). The membranes were probed with primary antibodies, including Flag (Proteintech, Wuhan, China, 66008-4-Ig), Myc (Proteintech, 60003-2-Ig), UBR5 (Proteintech, 66937-1-Ig), Snail (Santa Cruz Biotechnology, Oregon, USA, 166476), phosphorylated Snail (Biodragon, BD-PP0568), Slug (Santa Cruz Biotechnology, 271977), E-cadherin (Proteintech, 20874-1-AP), N-cadherin (BD Transduction Laboratories, Franklin Lakes, USA, 610920), GSK3β (Proteintech, 82061-1-RR), pGSK3β (Proteintech, 67558-1-Ig), green fluorescent protein (GFP; Proteintech, 66002-1-Ig), and glyceraldehyde-3-phosphate dehydrogenase (GAPDH; Bioss, Woburn, USA, 0978M).

For the immunoprecipitation assay, HEK293T cells were co-transfected with the indicated plasmids and treated with 10 μM proteasome inhibitor MG132 (Sigma–Aldrich, St. Louis, USA, C2211) for 6 h, cells were lysed, and the supernatants were incubated with RIPA binding buffer (150 mM NaCl, 50 mM Tris pH 7.5, 10% glycerol) and 20 μL of protein A/G agarose beads (Invitrogen, Carlsbad, USA, 20421) at 4 °C for 6 h. Immunoprecipitation was performed at 4 °C overnight using Flag (Proteintech, 66008-4-Ig) or Myc (Proteintech, 60003-2-Ig). The bead-antigen-antibody complexes were washed with RIPA buffer and analyzed by western blotting with the indicated antibodies.

### Immunofluorescence assay

An immunofluorescence assay was performed as previously described.^29^ Fluorescence phoography was captured with laser scanning confocal microscopy (Leica, LSM 710). The primary antibodies used in this experiment were UBR5 (Proteintech, 66937-1-Ig), Snail (Cell Signaling Technology, Danvers, USA, 3879S), and E-cadherin (Proteintech, 20874-1-AP). The secondary antibodies were CoraLite488-conjugated (Proteintech, SA00013-2), CoraLite594-conjugated (Proteintech, SA00013-3), and 4′,6-diamidino-2-phenylindole (DAPI; Beyotime, Shanghai, China).

### His-tag pull-down assay

Snail wild-type, truncated, and UBR5-truncated cDNAs were amplified by PCR and cloned into pET28a bacterial expression vectors with an N-terminal His-tag. These plasmids were transformed into BL2 (DE3) (TransGen, Beijing, China, CD601-02) or Transetta (DE3) (TransGen, CD801-02) cells. His-tagged Snail wild-type and truncated proteins were induced by 0.6 mM isopropyl β-d-1-thiogalactopyranoside (IPTG) at 37 °C for 5 h, and UBR5-truncated proteins by 0.4 mM IPTG at 21 °C for 16 h. His-tagged UBR5 truncated protein was induced by 0.4 mM IPTG at 21 °C for 16 h and purified using nickel-nitrilotriacetic acid (Ni-NTA) beads (QIAGEN, Hilden, Germany, 1018244).

HEK293T cells transfected with UBR5-Myc or Snail-Flag constructs were harvested in a lysis buffer (50 mM NaH_2_PO_4_.H_2_O pH 8, 300 mM NaCl, 10 mM imidazole, protease inhibitor cocktail) and sonicated. The cell lysates were incubated with purified His-tagged Snail or UBR5-truncated proteins and Ni-NTA beads. After washing with phosphate-buffered saline, bound proteins were eluted (50 mM NaH_2_PO_4_.H_2_O pH 8, 300 mM NaCl, 20 mM imidazole, protease inhibitor cocktail) and analyzed by western blotting using the indicated antibodies.

### Protein degradation assay

HEK293T cells were seeded in 6-well plates for 24 h and transfected with 0.5 μg of indicated expression plasmids. When indicated, 0.2 μg GFP was used as internal transfection control. After 16 h, transfected cells were treated with 10 μM MG132, 20 μM chloroquine (Medchemexpress, HY-17589) for 8 h, or 10 μM CT99021 (Aladdin, C408766) for 24 h. Following treatments, cells were lysed using ice-cold whole-cell extraction buffer (25 mM β-glycerophosphate, pH 7.3, 2 mM EGTA, 10 mM EDTA, 10 mM β-mercaptoethanol, 0.1 M NaCl, 1% Triton X-100, and a protease inhibitor cocktail), and protein lysates were prepared for western blotting analysis.

For concentration-dependent experiments, HEK293T cells were transfected with 2 μg of Snail-Flag or Snail 6SA-Flag, 0.2 μg of GFP conjugated with 0, 0.5, 1, 1.5, 2, 2.5 μg or UBR5-Myc-truncated and -mutated plasmids for 48 h. Cell lysates were immunoblotted with anti-Snail antibodies.

### *In vitro* ubiquitination assay

Wild-type Ub, Ub-K48, and Ub-K63 plasmids with HA tags were co-transfected with the relevant plasmids. After 18 h, transfected cells were added with 10 μM proteasome inhibitor MG132 for 6 h. The experimental manipulations were continued according to immunoprecipitation and western blotting analyses with indicated Flag and HA (Proteintech, 81290-1-RR) antibodies.

### Cycloheximide chase assay

HEK293T cells were seeded in six-well plates for 48 h and transfected with 1 μg of indicated expression plasmids and 0.2 μg of GFP as internal transfection control. After 24 h, transfected cells were treated with 50 μg/mL cycloheximide (Aladdin, Shanghai, China, C112766) for 0, 0.5, 1, 2, 4, and 6 h and collected in an ice-cold whole cell extraction buffer for western blotting analysis.

### Lentiviral short hairpin RNA (shRNA) depletion and quantitative reverse-transcription PCR

UBR5 expression was manipulated by infecting cells with lentiviral vectors for overexpression or shRNAs for knockdown. Lentiviral particles were produced in HEK293T cells using shRNA constructs and helper plasmids (psPAX2, pVSVG). After puromycin selection, RNA was extracted with TRIzol (Thermo Scientific, MA, USA), and cDNA was synthesized. Gene expression was quantified by real-time PCR and normalized to β-actin. Primers used for quantitative reverse-transcription PCR are listed in Table S1. The shRNA sequences are listed in Table S2.

### Wound-healing migration assay

Cells were seeded in 6-well plates and cultured to confluence. A wound was created by manually scraping the cell monolayer with a 200 μL tip. Cells were washed with phosphate buffer saline and maintained in a serum-free medium for the duration of the experiment. The wounds were photographed at 0 and 48 h following scraping, and the sizes of the healing were measured.

### Transwell invasion assay

Transwell chamber membranes (24-well; 8 μM, Costar, Corning, USA, 3422) were coated with fibronectin (Sigma–Aldrich). Cells (5 × 10^4^) were seeded into the top chamber and incubated at 37 °C overnight. Cells that had invaded the lower side of the membrane were fixed and stained with 1 % crystal violet (Solarbio, Beijing, China, G1062), imaged by microscopy, and counted.

### Mouse subcutaneous tumor formation assay

This study followed the NIH animal care guidelines and was approved by the Changchun Wish Technology Animal Ethics Committee. After stable expression of the corresponding plasmid cDNA in HCT116 cells infected with lentivirus, 5 × 10^6^ cells were collected. The cells were subcutaneously injected into the hindlimb of 4-to-6-week-old NOD/SCID mice. After 3 weeks, tumors were harvested, and the excised tumors were fixed with 4% paraformaldehyde, embedded in paraffin, and sectioned (thickness: 5 μM). Hematoxylin-eosin stained sections were observed and photographed under a microscope.

### Molecular docking simulations

We used the HDOCK SERVER (http://huanglab.phys.hust.edu.cn/) to perform protein–protein docking between the Snail C-zinc finger domain (amino acids 151–164) and the UBR5 HECT domain (amino acids 2453–2799). The top-ranked model (Model No. 1) was selected based on docking and confidence scores and further analyzed with PyMOL.

### Kaplan-Meier plot

We selected patient cohorts from the Kaplan–Meier Plotter website (http://kmplot.com/). Histological data from 165 patients with rectum adenocarcinoma were selected in pan-cancer RNA sequencing.

### Statistical analysis

UBR5 expression in CRC was analyzed using independent *t*-tests for unpaired samples and paired *t*-tests for paired samples. Categorical variables, including gender and tumor differentiation grade, were compared using chi-squared tests. All analyses were two-sided and conducted with R v.3.2.0 and SPSS v.16.0.2 (SPSS, Chicago, IL, USA). *P*-values <0.05 were considered statistically significant.

## Results

### UBR5 interacted with Snail

To gain deeper insights into Snail regulation and its biochemical activity, we used affinity purification-mass spectrometry to isolate Snail-interacting proteins. We identified multiple peptides derived from UBR5 (Fig. S1A). We used transcripts per million (TPM) analysis to evaluate the correlation between UBR5 and Snail expression in CRC. The analysis revealed a weak but statistically significant positive correlation in The Cancer Genome Atlas (TCGA) and GTEx databases (correlation coefficient: R = 0.29; *P* < 0.01; Fig. 1A). TNM stages II–III indicate that *in situ* cancer colonizes distant sites. Thus, we hypothesized that the EMT of tumor cells is activated in stage II, and the epithelial cells have transitioned to a mesenchymal morphology for migration to distant sites.^30^ Moreover, gene expression determines changes in the cell phenotype, as evidenced by low UBR5 and slightly elevated Snail expression in stage II disease (Fig. 1B). Combined with the clinical diagnosis of CRC tumors, we grouped the patients by high and low expression of UBR5. We found that low UBR5 expression was more frequently detected in stage II (Table 1). Overall, although transcriptomic data indicate a weak positive correlation between UBR5 and Snail expression, UBR5 was negatively correlated with Snail expression levels in stage II CRC, with low UBR5 expression and high Snail expression.

**Figure 1:**
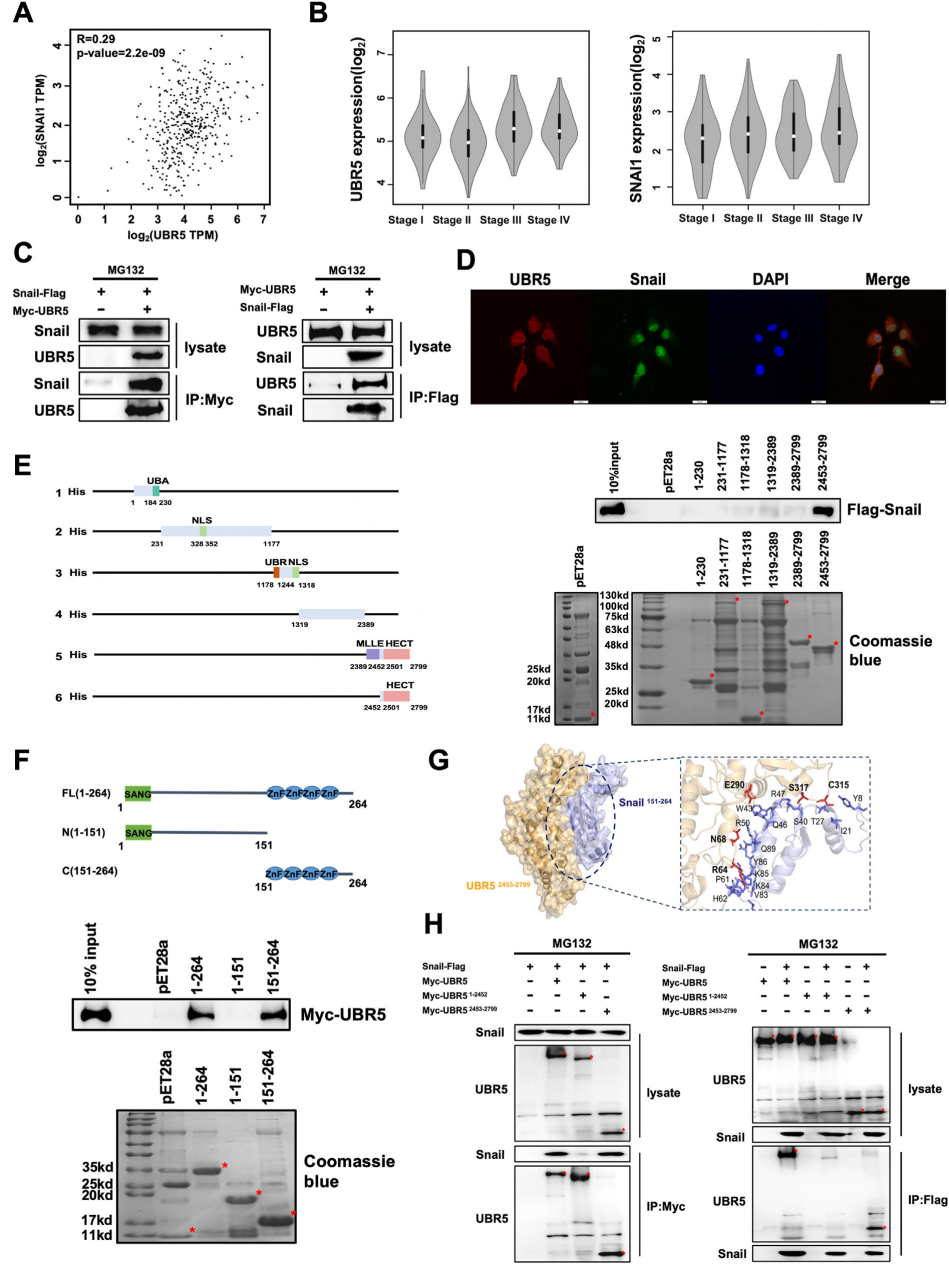
UBR5 interacted with Snail. **(A)** UBR5 was positively correlated with Snail in colon adenocarcinoma and rectal adenocarcinoma. Correlation analysis for TCGA and GTEx on the GEPIA website showed a correlation coefficient of R = 0.26, *P* = 4.4e-13. *P* < 0.01 denoted statistical significance. **(B)** The expression levels of UBR5 and Snail correlated with colorectal cancer (CRC) stages. The UBR5 and Snail mRNA levels based on pathological stages were analyzed using the GEPIA2 violin-plots in colorectal tumors. **(C)** Co-immunoprecipitation assay showed that UBR5 interacted with Snail. HEK293T cells were transfected with Myc-tagged UBR5 and Flag-tagged Snail and treated with MG132 as indicated. Cell lysates were immunoprecipitated with either anti-Myc or anti-Flag antibodies and immunoblotted with anti-Snail and anti-UBR5 antibodies. **(D)** Co-localization of UBR5 and Snail in the nucleus. Immunofluorescence assay probe co-localization of UBR5 (red) and Snail (green). Scale bar: 50 μm. **(E)** UBR5 interacted with Snail through the HECT domain. A schematic of various UBR5 truncations that are fused to His. Coomassie blue staining image of a PAGE gel, confirming the expression of pET28a and various UBR5 truncations. **(F)** Snail interacted with UBR5 through the zinc-figure domain. A schematic of various Snail truncations that are fused to His. Coomassie blue staining image of a PAGE gel, confirming the expression of pET28a and various Snail truncations. **(G)** Molecular docking of Snail zinc-figure domain (amino acids 151–264) and UBR5 HECT domain (amino acids 2453–2799) truncation protein. **(H)** The HECT domain of UBR5 interacted with Snail in the co-immunoprecipitation assay. HEK293T cells were transfected with wild-type and truncated Myc-tagged UBR5, as well as Flag-tagged Snail, and treated with MG132 as indicated. Cell lysates were immunoprecipitated with either anti-Myc or anti-Flag antibodies and immunoblotted with anti-Snail or anti-UBR5 antibodies.

**Table 1:** Correlation between UBR5 expression and clinicopathological factors in patients with colorectal cancer.

Characteristics	UBR5-High (*n* = 299)	UBR5-Low (*n* = 298)	*P*-value
Age (years), mean (SD)	64.38 (11.8)	67.15 (13.0)	0.007^a^
Sex (n (%))			0.905
Women	136 (45.5)	137 (46.0)	
Men	163 (54.5)	161 (54.0)	
Number of lymph nodes resected at surgery (n (%))			0.181
<12	58 (19.4)	47 (15.8)	
≥12	227 (75.9)	237 (79.5)	
Missing	14 (4.7)	14 (4.7)	
Lymphatic invasion			0.966
YES	103 (34.4)	107 (35.9)	
NO	161 (53.8)	166 (55.7)	
Missing	35 (11.7)	25 (8.4)	
Tumor stage			0.141^b^
i	57 (19.1)	50 (16.8)	
ii	87 (29.1)	127 (42.6)	
iii	92 (30.8)	77 (25.8)	
iv	52 (17.4)	34 (11.4)	
Missing	11 (3.7)	10 (3.4)	

∗*χ*^2^ test or Fisher's exact test.

a Student-*t* test.

b Mann–Whitney *U* test (non-parametric). Missing values are excluded for all statistic tests.

UBR5 is distinct from current E3 ligases of Snail, especially in regard to the substrate-recognition domains. Most E3 ubiquitin ligases that interact with Snail belong to the really interesting new gene (RING) family^31^; however, UBR5 is one of the HECT family. The potential association between UBR5 and Snail raised the possibility that UBR5 might act as a new ubiquitin ligase of Snail. To test this idea, we first verified the interaction between the two proteins and identified the specific domains involved. Co-immunoprecipitation with Flag-tagged Snail and Myc-tagged UBR5 in HEK293T cells showed that immunoprecipitation of Flag-tagged Snail pulled down Myc-tagged UBR5. In a reciprocal assay, Myc-tagged UBR5 also pulled down Flag-tagged Snail, confirming the interaction of UBR5 with Snail *in vitro* (Fig. 1C). The Snail family member Slug, also known as Snai2 and a core driver of EMT, was not pulled down by UBR5 in immunoprecipitation assays (Fig. S1B). Immunofluorescent staining of HCT116 colorectal cells showed that these proteins were localized in the nucleus (Fig. 1D). Mapping the key domains of UBR5 via truncations indicates that the HECT domain of UBR5 is responsible for interaction with Snail (Fig. 1E). To determine the part of Snail which binds to UBR5, Flag-tagged Snail truncations and pull-down experiments showed that the C-terminal end of Snail is essential for the interaction; loss of the C-terminal Zinc finger structural domain of Snail blocks interaction with UBR5 (Fig. 1F). These results were complemented by data obtained from molecular docking assays (Fig. 1G). Co-immunoprecipitation assays confirmed this interaction specifically with the HECT domain (amino acids 2453–2799) of UBR5 (Fig. 1H). These results suggest that UBR5 forms complexes with Snail in cells, and the binding domains identified are the UBR5 HECT domain and Snail Zinc figure domain.

### UBR5 promoted the degradation and polyubiquitination of Snail

Given UBR5's association with Snail, we hypothesized that UBR5 may function as the ubiquitin ligase responsible for Snail's degradation. First, we transfected cells with UBR5 to induce Snail degradation, this effect was blocked by cotreatment with the proteasome inhibitor MG132, but not by exposure to chloroquine (Fig. 2A). Furthermore, UBR5 did not accelerate the degradation of the Snail 6SA mutant, which blocks glycogen synthase kinase-3 beta (GSK-3β)-mediated phosphorylation and prevents Snail from exiting the nucleus (Fig. 2A; Fig. S2A). To further investigate the role of GSK3β, we treated cells with the GSK3β inhibitor CT99021 and observed that it effectively blocked UBR5-induced degradation of exogenous Snail (Fig. 2A). These findings collectively demonstrate that UBR5-mediated Snail degradation is dependent on GSK3β-mediated phosphorylation.

**Figure 2:**
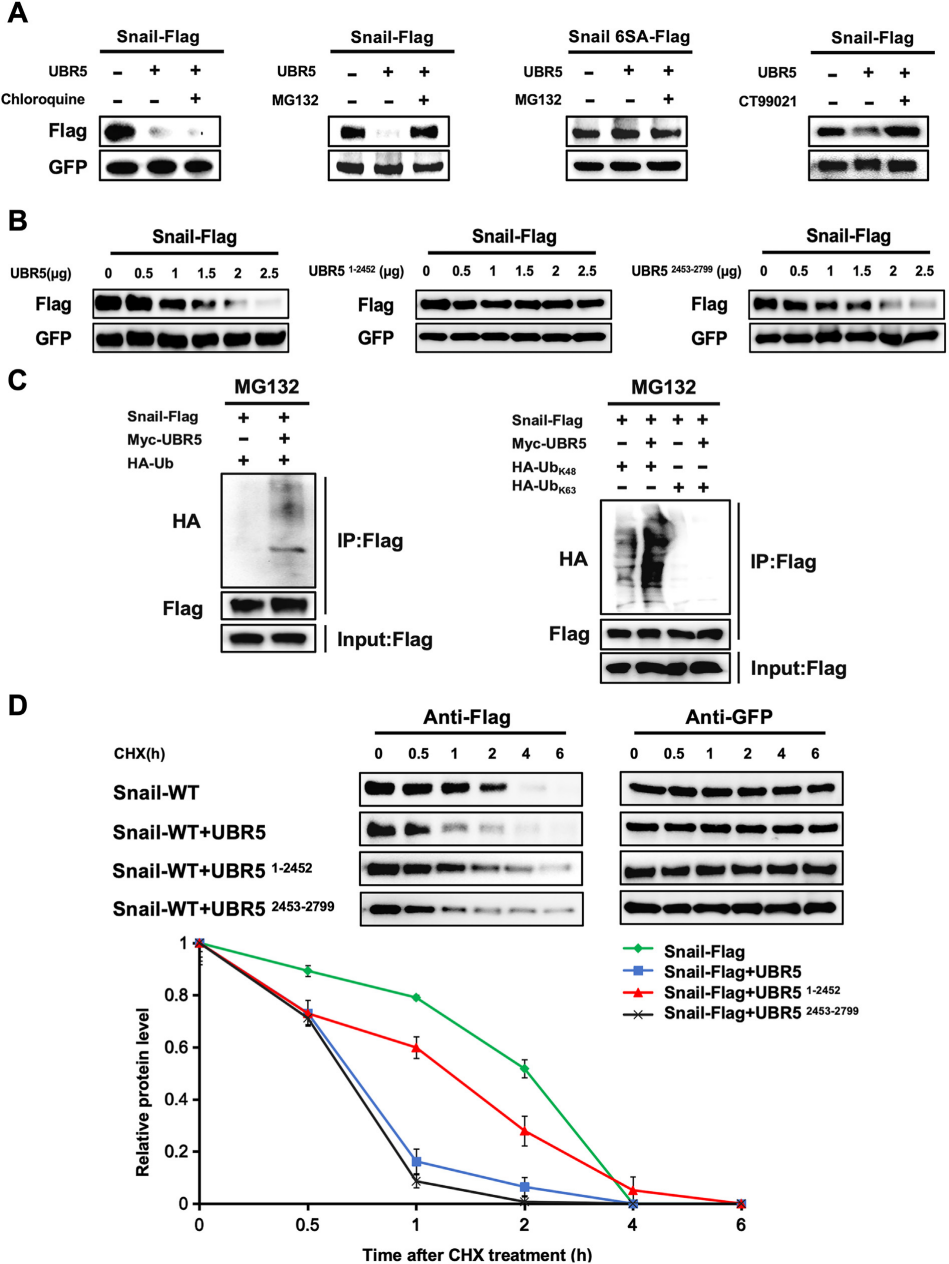
UBR5 promoted the degradation and polyubiquitination of Snail. **(A)** UBR5 promoted the proteasomal degradation of Snail. HEK293T cells were transfected with Snail-Flag, Snail 6SA-Flag, UBR5-Myc, GFP, or empty vector and treated with DMSO, chloroquine, MG132, or CT99021 as indicated. The expression of Snail and GFP was assessed by western blotting. **(B)** UBR5 degraded Snail protein in a concentration-dependent manner. HEK293T cells were transfected with Snail-Flag, GFP, or in combination with different concentrations of wild-type and truncated UBR5-Myc for 48 h. Cell lysates were immunoblotted with anti-Snail antibodies. **(C)** UBR5 promoted K48 polyubiquitinated chain generation of Snail protein. In cellular ubiquitination assays, UBR5-Myc were co-transfected with Snail-Flag plasmids or with HA-Ub-K63 and HA-Ub-K48 plasmids. Western blotting was performed on cell lysates immunoprecipitated with an anti-Flag antibody, followed by the detection of polyubiquitination levels using an anti-Ub antibody. **(D)** UBR5 accelerated the Snail protein turnover through the HECT domain. HEK293T cells were transfected with corresponding plasmids. Cells were treated with cycloheximide (CHX) and harvested at indicated time points for immunoblotting with anti-Snail or anti-GFP antibody. The graph shows the quantification of Snail protein levels (based on the band intensity from the gels) normalized to those of GFP over the time course. Snail protein expression at the 0 h time point of treatment with CHX was set as 100 %. Experiments were performed in triplicate, and a representative experiment is presented.

UBR5 modulates Snail degradation in a concentration-dependent manner (Fig. 2B). Since the HECT structural domain of UBR5 is indispensable for Snail binding, deletion of HECT affected UBR5's ability to degrade Snail (Fig. 2B). Overexpression of UBR5 markedly accelerated Snail ubiquitination, producing K48-linked ubiquitin chains (Fig. 2C). Snail is an unstable protein with a short half-life.^25^ To verify that UBR5 regulates Snail protein post-translationally via the ubiquitin-proteasome system, we inhibited Snail protein synthesis in HEK293T cells using cycloheximide. UBR5 accelerated the protein turnover of Snail but not Snail 6SA (Fig. 2D, Fig. S2B). Indeed, loss of the constitutive domain of HECT blocked the promotive effect of UBR5 on its half-life (Fig. 2D). Taken together, the results suggest that UBR5 selectively promotes polyubiquitination and degradation of Snail.

### UBR5 affected the expression of EMT-related factors

Snail plays a key role in driving EMT and directly represses epithelial gene expression in mesenchymal cells.^32^^,^^33^ UBR5's ability to down-regulate Snail prompted us to investigate its role in modulating the migration and invasion of CRC cells. We employed two independent lentiviral short hairpin RNAs (shRNAs) to down-regulate UBR5 in HCT116 cells, aiming to assess the expression of EMT-related genes. UBR5 down-regulation significantly up-regulated Snail protein levels while reducing E-cadherin expression (Fig. 3A). Additionally, the mesenchymal marker N-cadherin was elevated (Fig. 3A). The E-cadherin to N-cadherin switch is characteristic of EMT. The results of RNA level were basically consistent with the protein level (Fig. 3A). Similar to the *in vitro* results, down-regulation of endogenous UBR5 did not cause significant changes in slug protein and RNA levels in HCT116 cells, suggesting that there may be substrate specificity for UBR5 in CRC cells (Fig. 3A). Immunostaining results revealed that a subset of UBR5-down-regulated cells became Snail positive and E-cadherin negtive (Fig. 3B). Reduced E-cadherin expression may weaken intercellular adhesion, leading to altered cell morphology and behavior. As anticipated, UBR5-down-regulated HCT116 cells resulted in a predominance of dispersed, elongated single cells (Fig. 3C).

**Figure 3:**
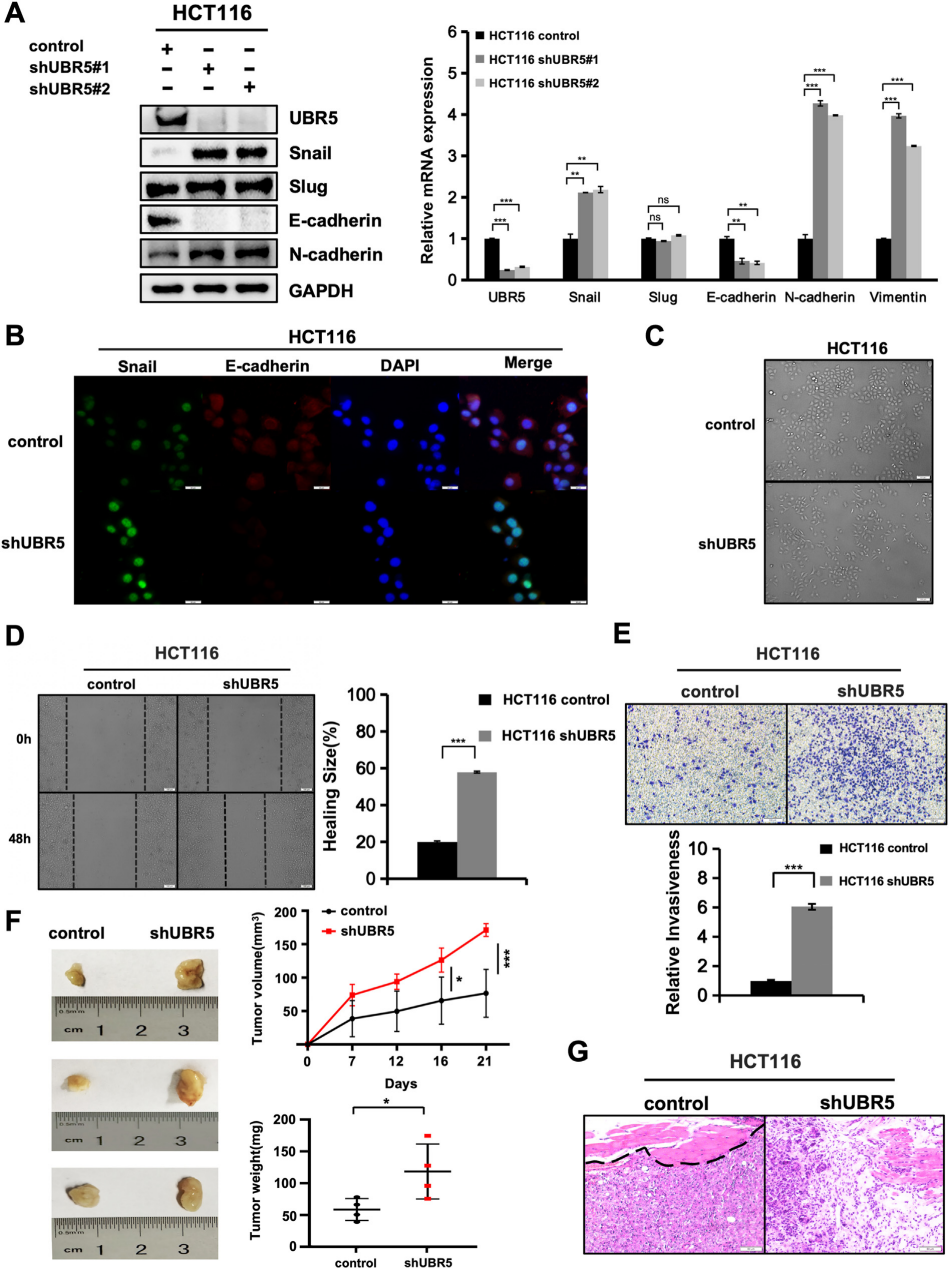
UBR5 affected the expression of epithelial–mesenchymal transition (EMT)-related factors. **(A)** Endogenous UBR5 knockdown changed the expression of Snail and EMT marker genes in colorectal cancer cells. Cells were collected and subjected to immunoblotting analysis and quantitative reverse transcription PCR analysis for indicated epithelial and mesenchymal markers. ∗*P* < 0.05, ∗∗*P* < 0.01, and ∗∗∗*P* < 0.001. **(B)** Immunofluorescence analysis of Snail and E-cadherin protein expression in control and shUBR5 of HCT116 cells (Snail, green; E-cadherin, red; DAPI, blue). Scale bar: 50 μm. **(C)** Depletion of UBR5 induced the EMT phenotype in colorectal cancer cells. Morphology of HCT116 cells after transfection with lentiviral shRNAs targeting either control or UBR5. Scale bar: 100 μm. **(D)** Reduction of UBR5 enhanced cell migration *in vitro*. Wound-healing experiments were performed to analyze changes in the migratory capacity of HCT116 control and shUBR5 cells. The histogram shows the quantitation of the relative degree of healing (*n* = 3). Scale bars: 100 μm. **(E)** Depletion of UBR5 facilitated cell invasiveness *in vitro*. Transwell assay was used to analyze changes in the invasive capacity of HCT116 control and shUBR5 cells. Scale bar: 100 μm. The number of cells crossing the basement membrane was counted. The histogram shows the quantitation of the relative numbers of cells that invaded and migrated through the matrix layer (*n* = 3). Scale bars: 100 μm. **(F)** Knockdown of UBR5 increased tumor volumes and weights. The photographs show the excised tumors from HCT116 control (left) and HCT116 shUBR5 (right) models (*n* = 3). The tumor sizes (tumor volumes and weights) were subjected to comparison. ∗*P* < 0.05 and ∗∗∗*P* < 0.001. **(G)** The knockdown of UBR5 promoted tumor cell infiltration. The effect on the xenograft model in HCT116 control and shUBR5 cells was assessed by hematoxylin-eosin staining. Scale bars: 50 μm.

To measure the migratory and invasive capacity of cells, the common *in vitro* wound-healing and transwell assays were conducted. UBR5-down-regulated cells exhibited enhanced motility, filling the gaps more rapidly than control cells in wound-healing assays (Fig. 3D). In addition, UBR5-down-regulated cells exhibited markedly higher trans-matrix migration in transwell assays, indicating a pronounced increase in invasive potential (Fig. 3E). Following down-regulation of UBR5, the similar results were also observed in other CRC cells examined, including SW480 cells (Fig. S3) and SW620 cells (Fig. S4). In an immunodeficient mouse xenograft tumor model, the knockdown of UBR5 cells significantly enhanced the growth and body weight of xenograft tumors compared with control HCT116 cells (Fig. 3F). Tumors in the control group, as shown by hematoxylin-eosin staining, exhibited well-defined boundaries and were encapsulated. In contrast, tumors with UBR5 knockdown demonstrated distinct signs of local invasion, including the infiltration of individual tumor cells into the surrounding muscle tissue (Fig. 3G). These findings underscore the crucial role of UBR5 in maintaining low Snail protein levels, thereby inhibiting EMT and preventing tumor invasion.

### C2768S mutation abrogated interaction with Snail

It was previously proposed that the C-lobe of UBR5 can retain its catalytic activity independently of the N-lobe, covalently binding ubiquitin. Mutation of the conserved cysteine residue at position 2768 in UBR5 to an alanine prevents the formation of thioesters.^22^^,^^34^ His pull-down assays indicated that the Cys2768 catalytic inactivation mutation in UBR5 is essential for the interaction of UBR5 with Snail proteins, highlighting the critical regulatory role of the HECT domain (Fig. 4A). This was further confirmed by co-immunoprecipitation and reciprocal co-immunoprecipitation. UBR5 C2768S mutation abolished the interaction between UBR5 and Snail (Fig. 4B; Fig. S5A). To directly test the effect of the C2768S mutation in UBR5 on Snail protein degradation, we co-transfected HEK293T cells with Snail-Flag, wild-type UBR5, or truncated and mutated forms of UBR5. We observed that exogenous Snail protein degradation was markedly increased by the UBR5 HECT truncated form. In contrast, UBR5 ΔHECT truncated (amino acids 1–2452) and UBR5 C2768S mutant lost their ability to affect Snail protein degradation (Fig. 4C; Fig. S5B, C). In a cycloheximide pulse-chase experiment, co-transfection with UBR5 accelerated Snail protein turnover. Nevertheless, the C2768S mutant did not, indicating that Cys2768 is a key amino acid in UBR5-Snail interaction (Fig. 4D). Taken together, these results demonstrate that UBR5, a potential E3 ubiquitin ligase, is involved in Snail protein homeostasis *in vitro* and that the C2768S mutation prevents this regulation.

**Figure 4:**
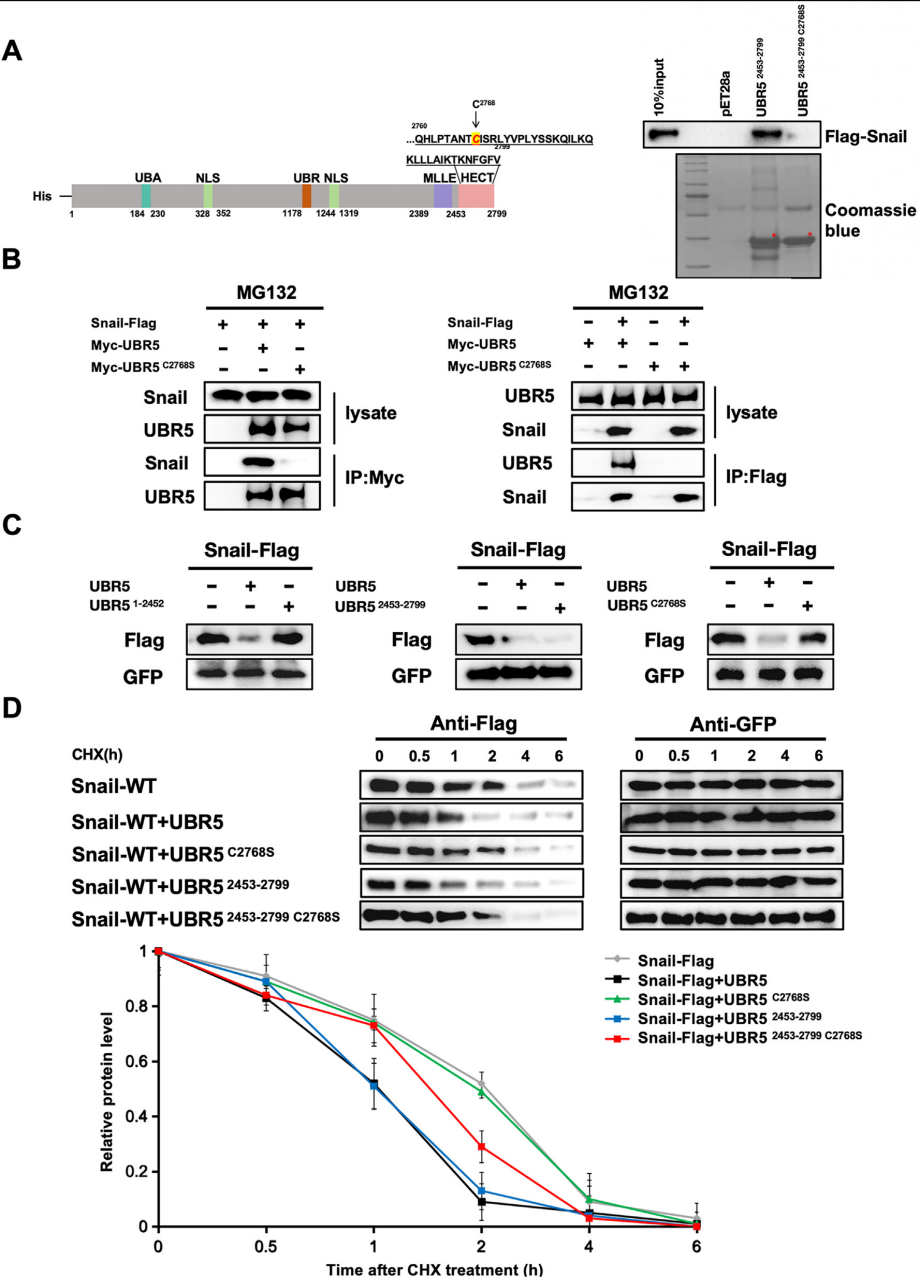
UBR5 C2768S mutation abrogated the interaction with Snail. **(A)** His pull-down assays showed the abolished interactions between Snail and the UBR5 C2768S. A schematic representation of the UBR5 wild-type and C2768S mutation. **(B)** Co-immunoprecipitation assay showed that the interaction between the Snail and the UBR5 C2768S mutation was eliminated. HEK293T cells were transfected with UBR5-Myc, UBR5 C2768S-Myc, and Snail-Flag as indicated. Cell lysates were immunoprecipitated with either anti-Myc or anti-Flag antibodies and immunoblotted with anti-Snail and anti-UBR5 antibodies. **(C)** UBR5 C2768S abolished the UBR5-mediated degradation of Snail. HEK293T cells were transfected with Snail-Flag, UBR5-Myc, and UBR5 C2768S-Myc as indicated. Cell lysates were subjected to western blotting analysis with anti-Snail and anti-GFP antibodies. **(D)** UBR5 C2768S did not accelerate Snail protein turnover. HEK293T cells were transfected with Snail-Flag, UBR5-Myc, and UBR5 C2768S-Myc and treated with cycloheximide (CHX) as indicated. Cell lysates were subjected to western blotting analysis with anti-Snail and anti-GFP antibodi.

### C2768S mutation eliminated UBR5's ability to regulate the migration and invasion of HCT116 cells

To test the functional impact of UBR5 C2768S mutant on Snail-induced EMT in CRC metastasis, we generated stable cell lines overexpressing UBR5 and the UBR5 C2768S mutant. As expected, the overexpression of UBR5 in HCT116 cells suppressed endogenous Snail protein and mRNA expression, blocking Snail-induced changes in EMT-related genes (Fig. 5A). In contrast, the reduction in Snail expression and the observed alterations in the expression of E-cadherin, N-cadherin, and vimentin were nullified in the UBR5 C2768S mutant cell line, in which the expression levels paralleled those measured in the control group (Fig. 5A). The results of immunofluorescence were basically consistent with those of protein and mRNA (Fig. 5B). These findings indicated that the C2768S mutation in UBR5 affects the regulation of E-cadherin in HCT116 cells.

**Figure 5:**
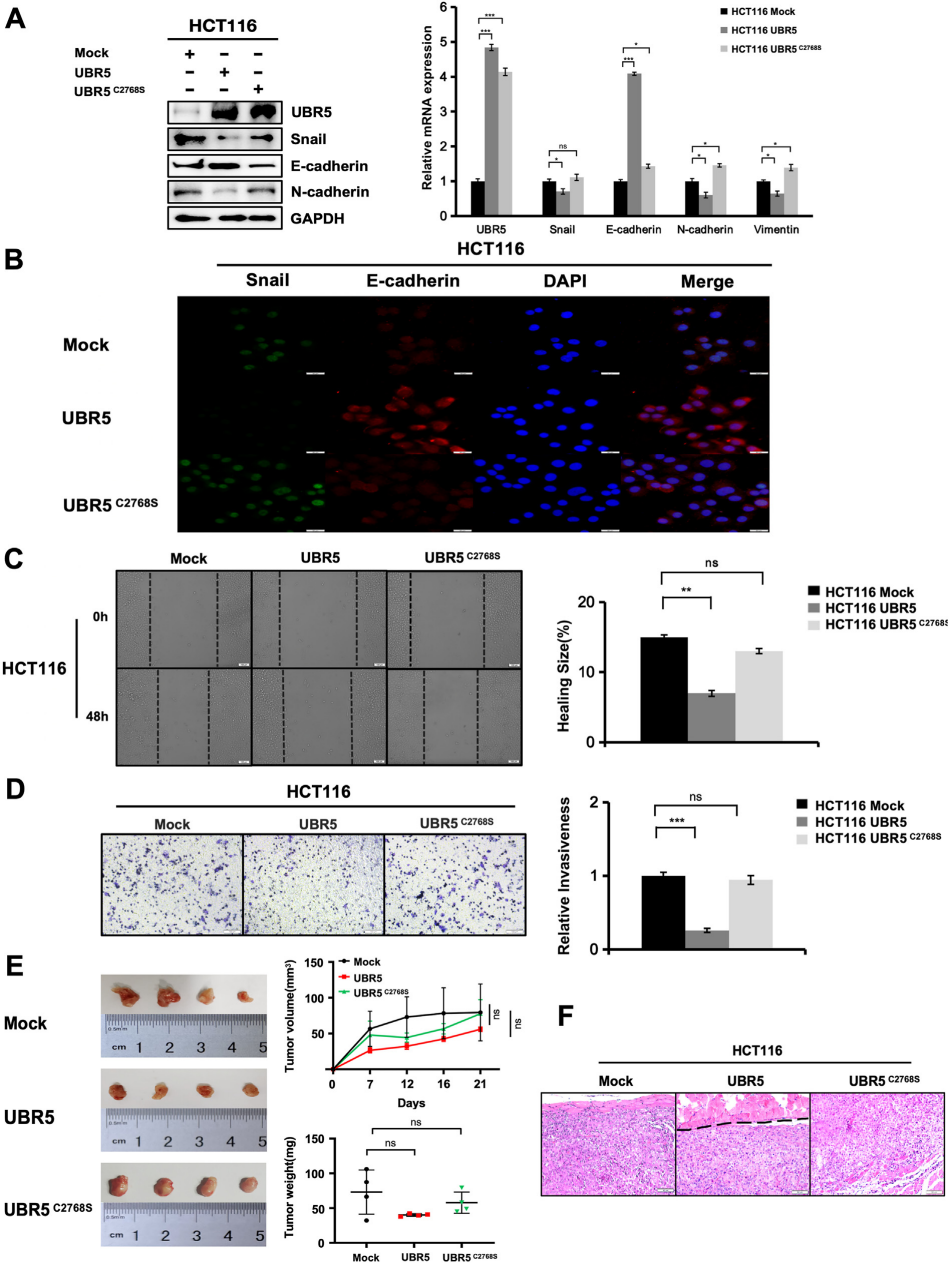
C2768S mutation abolished the effects of UBR5 on the migration and invasion of HCT116 cells. **(A)** C2768S mutation changed the expression of epithelial–mesenchymal transition marker genes. HCT116 cells were transfected with UBR5-Myc and UBR5 C2768S-Myc constructs. Cells were collected and subjected to immunoblotting analysis and quantitative reverse transcription PCR analysis for indicated epithelial and mesenchymal markers. ∗∗*P* < 0.01 and ∗∗∗*P* < 0.001. **(B)** Immunofluorescence analysis of Snail and E-cadherin protein expression in Mock, UBR5-Myc, and UBR5 C2768S-Myc in HCT116 cells (Snail, green; E-cadherin, red; DAPI, blue). Scale bar: 50 μm. **(C)** Wound-healing assays showed the migration of HCT116 cells transfected with Mock, UBR5-Myc, or UBR5 C2768S-Myc. Representative images of healing degrees at 0 and 48 h after performing the wound are shown. The histogram shows the quantitation of the relative healing degrees (*n* = 3). Scale bars: 100 μm. **(D)** Transwell assays showed the invasiveness of HCT116 cells transfected with Mock, UBR5-Myc, or UBR5 C2768S-Myc. Representative images of the staining of the cells that invaded and migrated through the matrix layer are shown. The histogram shows the quantitation of the relative numbers of cells that invaded and migrated through the matrix layer (*n* = 3). Scale bars: 100 μm. **(E)** Wild-type UBR5 tumors were smaller in volume and weight than the HCT116 Mock and C2768S mutant groups. The photographs show the excised tumors from HCT116 Mock, UBR5, and UBR5 C2768S models (*n* = 4). The tumor sizes (tumor volumes and weights) were subjected to comparison. **(F)** The UBR5 C2768S mutation disrupted the UBR5-Snail axis, eliminating its regulatory effect on tumor cell invasion. Hematoxylin-eosin staining of xenograft tumors derived from HCT116 Mock, UBR5-Myc, and UBR5 C2768S-Myc cells. Scale bar: 50 μm.

Based on gene expression changes, we functionally tested the effect of the C2768S mutation on tumor invasiveness. In wound-healing assay, cells transfected with the wild-type UBR5 displayed decreased motility, but transfection of the UBR5 C2768S mutant did not have significant influences on the migration of HCT116 cells (Fig. 5C). For transwell assay, HCT116 cells showed high invasion capability (Fig. 5D). While HCT116 cells transfected with wild-type UBR5 showed minimal invasion through the matrix, those transfected with the UBR5 C2768S mutant had a similar number of invaded cells as the control group, indicating that the mutant did not suppress the invasive capacity of HCT116 cells (Fig. 5D). In the immunodeficient mouse xenograft tumor model, the tumors formed by HCT116 cells stably transfected with wild-type UBR5 had the smallest volume and weight compared with both the control and C2768S mutant UBR5 groups, where tumor volume and weight were similar (Fig. 5E). When xenografted into immunodeficient mice, tumors from HCT116 cells stably transfected with wild-type UBR5 formed well-encapsulated masses with clear boundaries and showed less invasion into the adjacent muscle fibers (Fig. 5F). In contrast, tumors from HCT116 cells transfected with C2768S mutant UBR5 showed greater infiltration into the surrounding muscle fibers, similar to the control cells (Fig. 5F). This suggests that C2768S mutant UBR5 cells have a higher invasive potential compared with wild-type UBR5 cells. Collectively, these results demonstrated that the C2768S mutation eliminated UBR5's ability to regulate the migration and invasion of HCT116 cells.

### UBR5 was a favorable prognostic factor in human CRC

**Figure 6:**
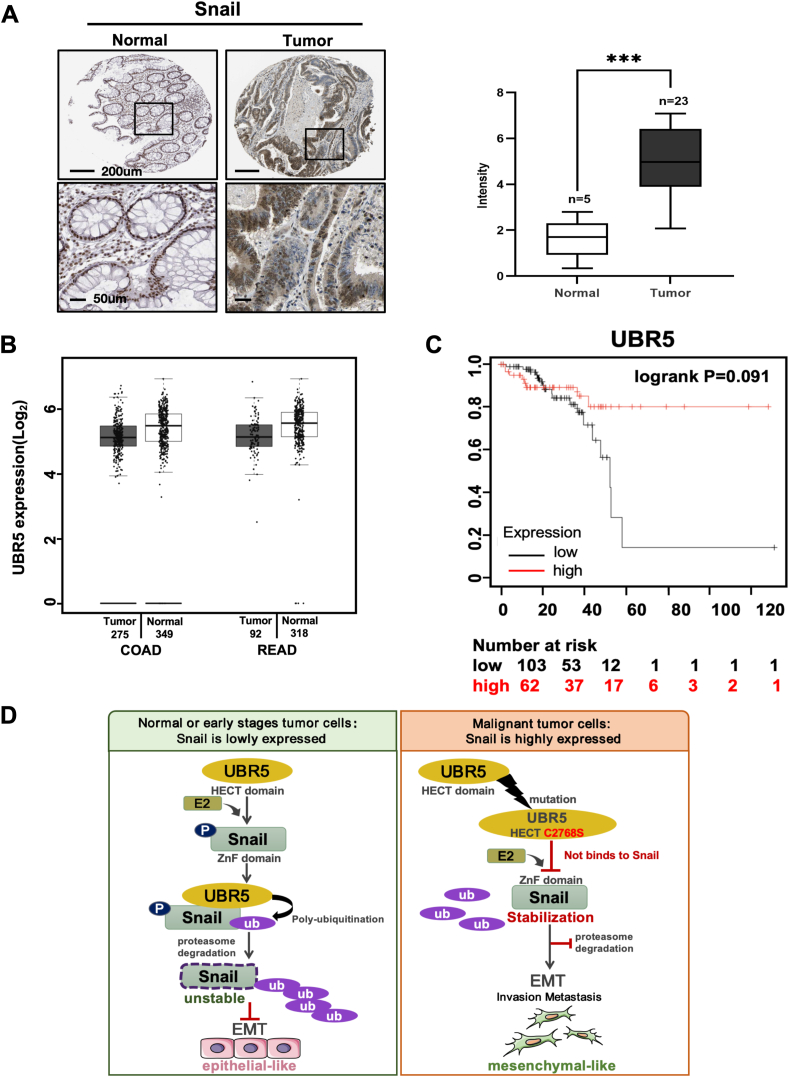
UBR5 was a favorable prognostic factor in human colorectal cancer. **(A)** Snail was highly expressed in samples obtained from patients with colorectal cancer. Immunohistochemical analysis of Snail expression levels in normal colorectum and tumors in the Human Protein Atlas website. Representative images are shown. ∗∗∗*P* < 0.001; student's *t*-test. **(B)** GEPIA revealed that UBR5 had higher expression in normal tissue samples compared with tumor samples. Dark and light gray indicate tumor and normal tissues, respectively. **(C)** High expression of UBR5 was associated with a favorable prognosis. Kaplan–Meier analysis of 20-year overall survival of rectum adenocarcinoma cancer patients with UBR5 (*n* = 165). Log-rank *P*-values are shown. **(D)** Diagram of the pattern of UBR5 regulation of epithelial–mesenchymal transition (EMT) in colorectal cancer. UBR5 inhibited the invasive migration of tumor cells by regulating the ubiquitination and transcriptional activity of Snail, and UBR5 C2768S eliminated the inhibitory effect of UBR5 on EMT.

To assess the practical value of the UBR5-Snail axis in CRC, we analyzed a series of human cancer sample databases. The results of immunohistochemical analysis showed that Snail was highly expressed in samples obtained from patients with CRC (https://www.proteinatlas.org) (Fig. 6A). We investigated UBR5 expression in normal and cancer tissues in The Cancer Genome Atlas and GTEx databases (http://gepia.cancer-pku.cn). The findings showed that UBR5 was lowly expressed in CRC tissues versus normal tissues (Fig. 6B). In rectal adenocarcinoma, consistent with its role in promoting Snail protein degradation, higher UBR5 expression was correlated with longer relapse-free survival (Fig. 6C). These results are consistent with our *in vitro* and *in vivo* experimental findings. In summary, UBR5 significantly interacts with Snail in CRC, and UBR5 can ubiquitinate Snail and target it for degradation by the proteasome, thereby inhibiting the EMT process. Site 2768 of the UBR5 HECT structural domain plays a key role in the binding and subsequent regulation of Snail. Lower UBR5 expression correlates with cancer progression, while higher expression is associated with a better prognosis (Fig. 6D).

## Discussion

As a leading cause of cancer-related mortality, CRC is responsible for a significant health burden worldwide.^34^^,^^35^ CRC often progresses to an advanced stage before detection, leading to poor prognosis and survival rates. During tumorigenesis and metastasis, cancer cells undergo EMT and mesenchymal–epithelial transition, enabling migration, invasion, and secondary tumor formation, influenced by the tumor microenvironment, genetic changes, and metabolic reprogramming. These transitions enable cancer cells to adapt to changing environments by modifying cellular processes, enhancing survival through DNA repair, immune evasion, and resistance to chemotherapy and radiotherapy.^16^^,^^36^ However, distinguishing changes in the expression levels of key targets from those induced by intrinsic EMT is currently a major challenge in the clinical diagnosis of tumors.

Therefore, identifying integrated targets that accurately reflect the lesion status remains critical. EMT is regulated by multiple factors, including the Snail, Twist, and ZEB families, which suppress epithelial genes while promoting mesenchymal gene expression. Recent studies have primarily concentrated on single-function mechanisms.^37^^,^^38^ Abnormal Snail expression has been observed in various carcinomas and is linked to malignant processes, including increased cell motility, proliferation, senescence, and apoptosis.^39^ In a *Drosophila* model of CRC metastasis, Snail was employed to induce EMT in a benign CRC model,^40^ producing results similar to those observed in human CRC. Based on the data, Snail, an indispensable driver of EMT, can serve as a consistent factor in the cancer process and offer potential for subsequent combination therapy.

Our study innovatively demonstrated that the E3 ubiquitin ligase UBR5 counteracted invasive tumor migration by inhibiting Snail-driven EMT via the ubiquitin-proteasome pathway. UBR5 interacted with Snail through a specific binding domain and restored Snail protein expression in MG132-treated cells. Overexpression of UBR5 increased the ubiquitination and mediated the K48-linked polyubiquitination of Snail, thereby regulating its protein stability in a concentration-dependent manner. Interestingly, the regulation of Snail by UBR5 is dependent on phosphorylation. The activity-deficient mutant UBR5 C2768S eliminated the modulation of Snail by UBR5, highlighting the specificity of this interaction. In CRC cell lines, UBR5 knockdown resulted in a more mesenchymal cellular morphology, elevated Snail expression, increased E-cadherin, and decreased N-cadherin and vimentin. Conversely, UBR5 overexpression inhibited Snail expression. Our *in vivo* mouse model showed that UBR5 knockdown promoted the role of Snail in tumor growth, peripheral muscle tissue infiltration, and distal metastasis. This suggests that regulation of the UBR5-Snail axis could counteract tumorigenesis and metastasis in CRC.

Based on our finding that UBR5 cannot degrade the Snail-6SA mutant, we speculate that the action of UBR5 on Snail requires Snail phosphorylation by GSK-3β before it is transferred from the nucleus to the cytoplasm, where it can interact with UBR5. Similar results were observed in CRC cell lines. In HCT116 cells, UBR5 overexpression significantly reduced total Snail protein levels and destabilized phosphorylated Snail. However, inhibiting GSK3β with CT99021 effectively restored Snail stability, highlighting the essential role of GSK3β-driven phosphorylation in this regulatory axis (Fig. S6A). Further validation was obtained in shGSK3β knockdown HCT116 cells, where UBR5 overexpression failed to promote Snail degradation, reinforcing the critical role of GSK3β in mediating UBR5 activity (Fig. S6B, C).

Additionally, UBR5 cannot affect Slug in CRC cells, indicating substrate specificity, and we explored the selectivity and effectiveness of E3 ubiquitin ligases in therapy by combining large protein targets and PROTAC technology.^41^^,^^42^ Future studies should focus on elucidating the *in vivo* role of the UBR5-Snail axis in CRC and exploring the complex interactions between EMT regulators and other signaling pathways in CRC to gain a comprehensive understanding of tumor progression. The wide-ranging effects of UBR5 in other cancer types may provide valuable insights into its role as an E3 ubiquitin ligase. Understanding the subcellular localization of UBR5 and its interactions with different substrates will assist in revealing its multifaceted functions. The results of our study highlight the importance of research on early cancer to support early diagnostic and therapeutic strategies and have important implications for the development of E3 ubiquitin ligase-based therapeutics. This evidence provides a theoretical basis for the selection and optimization of these technologies.

## CRediT authorship contribution statement

**Xinyue Zhao:** Writing – original draft, Methodology, Investigation, Data curation. **Ruiying Liu:** Software, Formal analysis. **Zhihui Han:** Visualization, Data curation. **Zehao Li:** Resources. **Ling Mei:** Validation. **Yuyang Liu:** Software. **Xueqi Fu:** Supervision. **Yue Jin:** Writing – review & editing, Project administration, Funding acquisition, Conceptualization.

## Ethics declaration

The animal study protocol was approved by the Ethics Committee of the Changchun Wish Technology Company (Approved Number: 20240128-01, approved on 19 January 2024).

## Data availability

The datasets used and analyzed during the current study are available from the corresponding author upon reasonable request.

## Funding

This work was supported by the Jilin Provincial Scientific and Technological Development Program (China) (No. 20220101276JC, 20210402018GH) and the National Key R & D Program of China (No. 2021YFA1500403).

## Conflict of interests

The authors declared no conflict of interests.

## Figures and Tables

**figs1:**
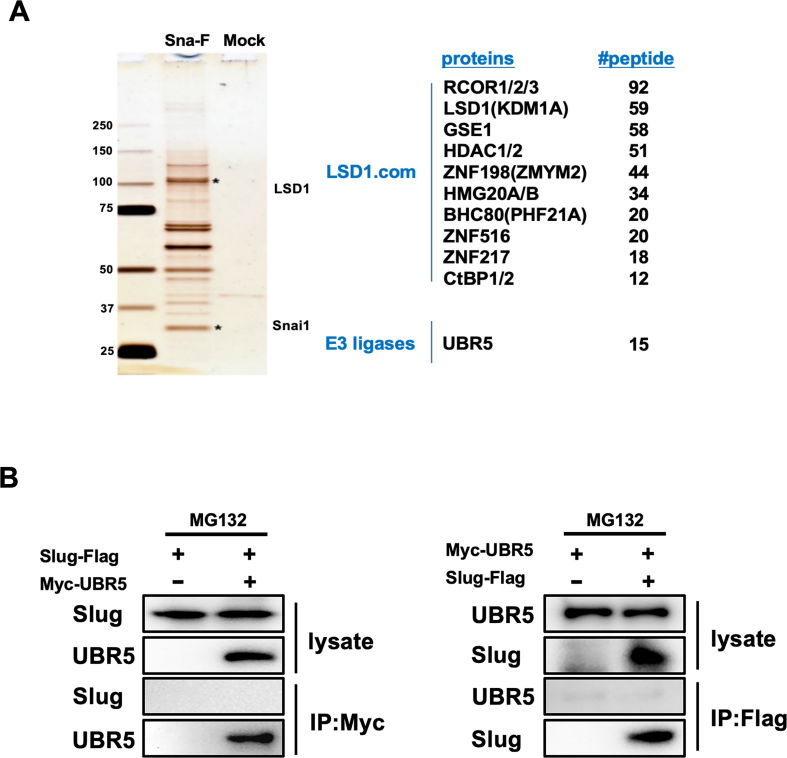


**figs2:**
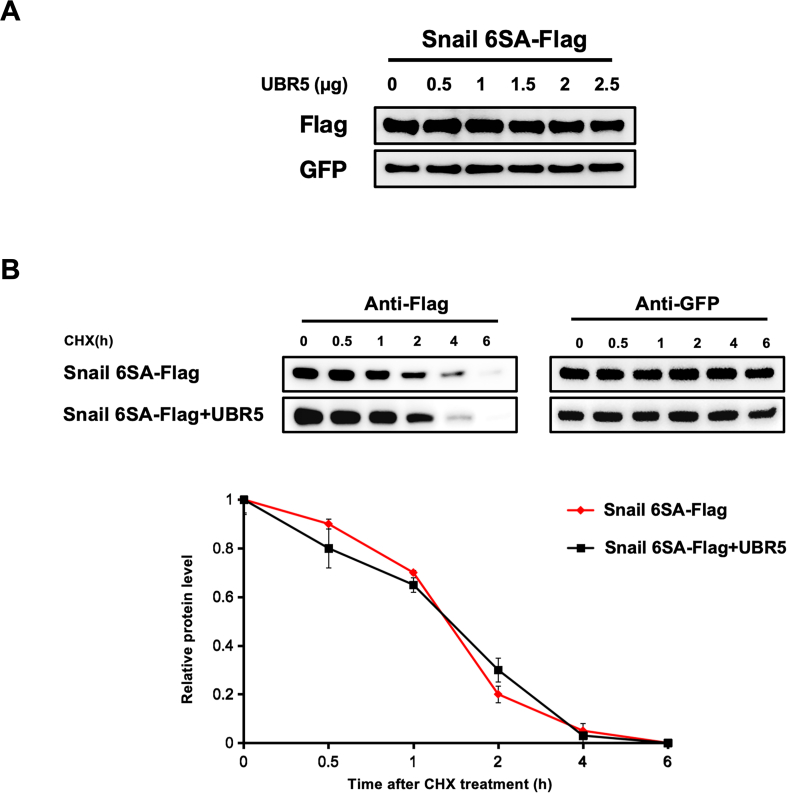


**figs3:**
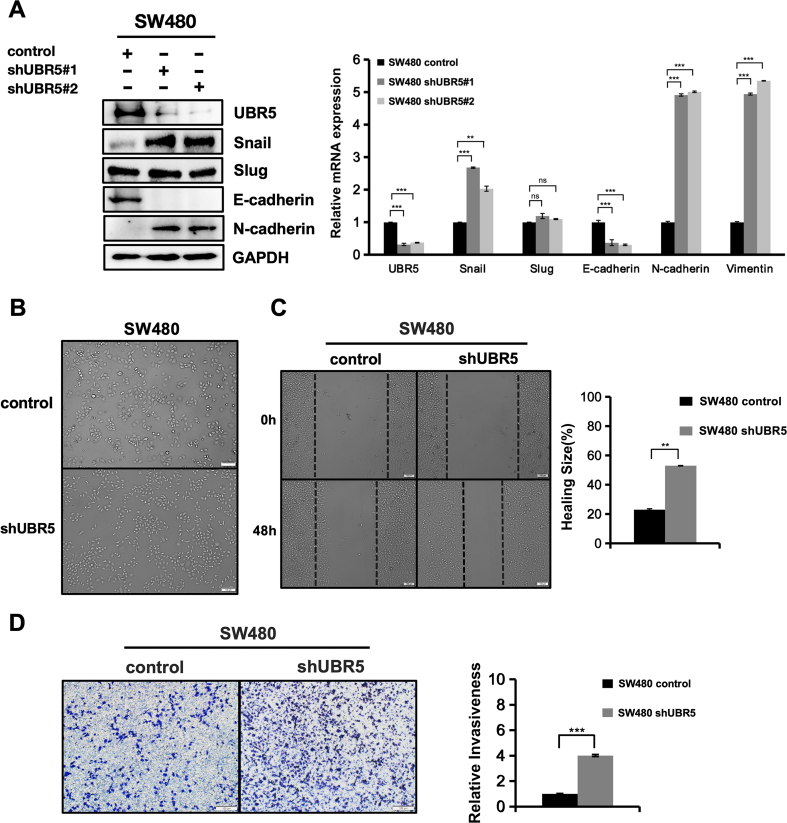


**figs4:**
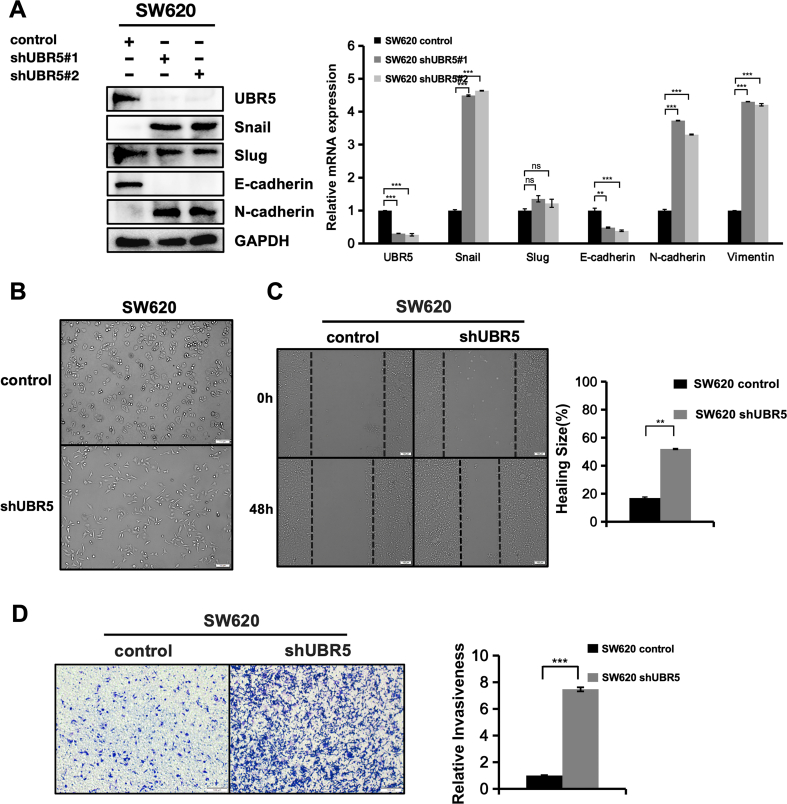


**figs5:**
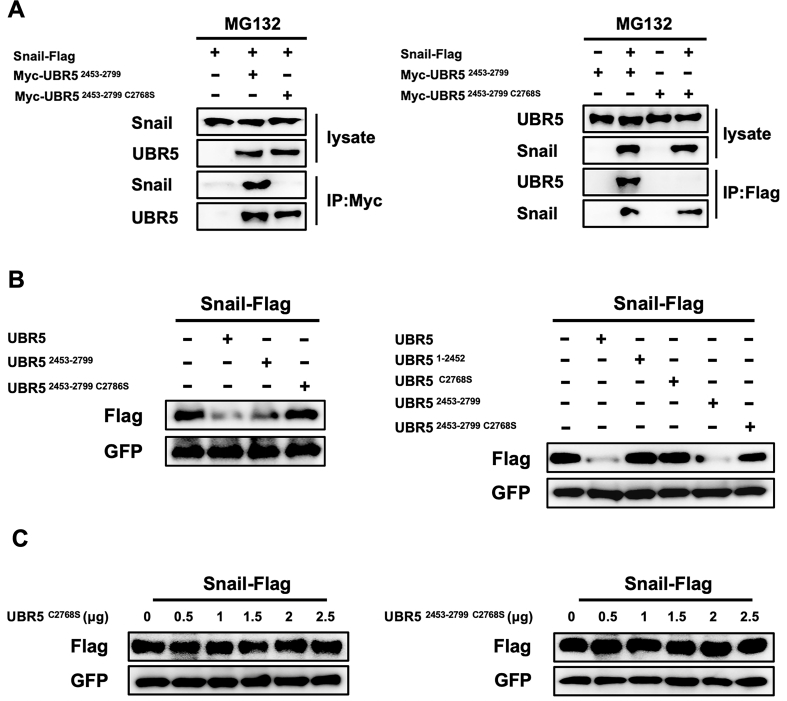


**figs6:**