# Supplementary material for: UBR5 regulates the progression of colorectal cancer cells through Snail-induced epithelial–mesenchymal transition
Source: Genes Dis. 2025 May 13;13(2):101679. doi: 10.1016/j.gendis.2025.101679 (PMC12765265; doi:10.1016/j.gendis.2025.101679)
Supplement: Multimedia component 1 [file mmc1.docx]

**Supplementary Table1**

q-PCR primers

| Primers | 序列 |
| --- | --- |
| UBR5：  SNAIL1：  SNAIL2:  CDH1:  CDH2:  VIM :  β-actin： | Forward: TTAGGCTTTTGGTAAATGGCTGCG  Reverse: TGAGGGCATAGGCTGGAATCCTTC  Forward: TGGTTCTTCTGCGCTACTGC  Reverse: GCTGCTGGAAGGTAAACTCTGG  Forward: CTTCCTGGTCAAGAAGCA  Reverse: GGGAAATAATCACTGTATGTGTG  Forward: CAGTGAACAACGATGGCATT  Reverse: CTGGGCAGTGTAGGATGTGA  Forward: TCAGGCTCCAAGCACCCCTTCA  Reverse: ATGACGGCCGTGGCTGTGTT  Forward: ATTGAGATTGCCACCTACAG  Reverse: ATCCAGATTAGTTTCCCTCAG  Forward: AGAAAATCTGGCACCACACC  Reverse: AGAGGCGTACAGGGATAGCA |

**Supplementary Table2**

Lentiviral packaging sequence

| shRNA seq |  |
| --- | --- |
| shUBR5#1 | GATAAAGTAGATCAAGACGAGC |
| shUBR5#2 | GTTTAATAGTAGCCTGTTCCAA |
| shGSK3β#1  shGSK3β#2 | GACACTAAAGTGATTGGAAAT  GTGTGGATCAGTTGGTAGAAA |
