# Supplementary material for: UBR5 regulates the progression of colorectal cancer cells through Snail-induced epithelial–mesenchymal transition
Source: Genes Dis. 2025 May 13;13(2):101679. doi: 10.1016/j.gendis.2025.101679 (PMC12765265; doi:10.1016/j.gendis.2025.101679)
Supplement: Multimedia component 2 [file mmc2.docx]

**Supplementary Fig. S1**

1. Identification of UBR5 as a Snail-associated protein through mass spectrometry. Key reproducible peptides are shown.
2. Co-IP assay showed that UBR5 not interacted with Slug. HEK293T cells were transfected with Myc-tagged UBR5 and Flag-tagged Slug, as indicated. Cell lysates were immunoprecipitated with either anti-Myc or anti-Flag antibodies and immunoblotted with anti-Slug and anti-UBR5 antibodies.

**Supplementary Fig. S2**

(A) UBR5 did not degrade Snail 6SA protein in a concentration-dependent manner. HEK293T cells were transfected with Snail 6SA-Flag, GFP, or in combination with different concentrations of wild-type UBR5-Myc for 48 h. Cell lysates were immunoblotted with anti-Snail and anti-GFP antibodies.

(B) UBR5 did not exert an effect on the half-life of Snail 6SA. HEK293T cells were transfected with Snail 6SA-Flag and UBR5-Myc, and treated with CHX as indicated. Cell lysates were subjected to western blotting analysis with anti-Snail and anti-GFP antibodies.

**Supplementary Fig. S3**

(A) Endogenous UBR5 knockdown in SW480 cells stabilized Snail protein and changed the characteristic gene expression of EMT. SW480 cells were transfected with control and shUBR5. Subsequently, the cells were collected and subjected to western blotting analysis and RT-qPCR analysis for indicated epithelial and mesenchymal markers.

(B) SW480 cells were transfected with lentiviral shRNAs targeting control or UBR5. Images of control and UBR5-depleted cells showing morphological differences. Scale bar: 100 μm.

(C) Wound-healing assay showing the migration of SW480 cells transfected with control and shUBR5. Representative images are shown at 0 and 48 h after performing the wound (n=3). Scale bar: 100 μm.

(D) Transwell assay showing the invasiveness of SW480 cells transfected with control and shUBR5 (n=3). Scale bar: 100 μm.

**Supplementary Fig. S4**

(A) Endogenous UBR5 knockdown in SW620 cells stabilized Snail protein and changed the characteristic gene expression of EMT.

(B) SW620 cells were transfected with lentiviral shRNAs targeting control or UBR5. Images of control and UBR5-depleted cells showing morphological differences. Scale bar: 100 μm.

(C) Wound-healing assay showing the migration of SW620 cells transfected with control and shUBR5. Representative images are shown at 0 and 48 h after performing the wound (n=3). Scale bar: 100 μm.

(D) Transwell assay showing the invasiveness of SW620 cells transfected with control and shUBR5 (n=3). Scale bar: 100 μm.

**Supplementary Fig. S5**

(A) The HECT domain of the 2768 site in UBR5 played an important role in the interaction with Snail. HEK293T cells were transfected with UBR5^2453-2799^-Myc, UBR5^2453-2799 C2768S^-Myc, and Snail-Flag, and treated with MG132 for 6 h before lysis. Whole-cell extracts were subjected to co-IP with anti-Myc or anti-Flag antibody, and immunoblotting with indicated antibodies.

(B) UBR5 C2768S did not promote Snail degradation. HEK293T cells were transfected with Snail-Flag, together with or without UBR5 wild-type and UBR5 C2768S mutation together with GFP as well as empty vector. Cell lysates were immunoblotted with indicated antibodies.

(C) UBR5 C2768S mutation did not degrade Snail protein in a concentration-dependent manner. HEK293T cells were transfected with Snail-Flag, GFP, or in combination with different concentrations of UBR5 C2768S mutation, or UBR5^2453-2799 C2768S^ mutation for 48 h. Cell lysates were immunoblotted with indicated antibodies.

**Supplementary Fig. S6**

1. Treatment with the CT99021 rescued Snail protein stability in the presence of UBR5 overexpression. Immunoblotting analysis was performed on HCT116 cells transfected with control or UBR5 plasmids to evaluate total Snail and pSnail levels.
2. Validation of GSK3β knockdown efficiency in HCT116 cells. HCT116 cells were transfected with shGSK3β or control shRNA. Immunoblotting analysis confirmed the efficient depletion of GSK3β protein.
3. GSK3β knockdown disrupted UBR5-mediated degradation of Snail and pSnail. HCT116 cells overexpressing UBR5 were transfected with shGSK3β or control. Immunoblotting analysis for indicated markers.
