# Supplementary material for: UBR5 regulates the progression of colorectal cancer cells through Snail-induced epithelial–mesenchymal transition
Source: Genes Dis. 2025 May 13;13(2):101679. doi: 10.1016/j.gendis.2025.101679 (PMC12765265; doi:10.1016/j.gendis.2025.101679)
Supplement: Multimedia component 3 [file mmc3.pdf]

实验动物福利伦理审查表

Application Format for Ethical Approval for Research Involving Animals

申请日期： 2024 年 1 月 19 日　　受理编号：20240122-01　　批准文号：20240128-01

Appl. Date 2024 Y 1 M 19 D　　Appl. No 20240122-01　　IACUC Issue No. 20240128-01

课题或实验名称及编号：UBR5 泛素化 Snail 调控结直肠癌细胞 EMT 的分子机制<br>Program and No.：Molecular mechanism of UBR5 ubiquitinated Snail regulation of EMT in colorectal cancer cells	课题来源：吉林省科技发展计划国际合作项目<br>Sponsor：Jilin Science and Technology Development Program for International Cooperation
课题负责人 金悦<br>Name of Principal Investigator Yue Jin	单位 生命科学学院 Fisher 实验室<br>Department：College of Life Science，Fisher Laboratory
动物实验负责人 赵昕月<br>Contact Person Xinyue Zhao	电话 Contact Tel. No. 16688207020<br>信箱 Email：zhaoxinyue199711@163.com
课题实施动物实验的人数 2<br>Number of Implement Two	经专业培训的人数 2<br>Number of Certificate Two

参与动物实验操作人员姓名、相关专业证书编号， 经验、 培训 、资质和能力的描述

Name and certificate number , Description of experience/ training/competency of the individuals carrying out the research.

赵昕月，在吉林大学生命科学学院受过动物实验上岗资格及动物福利伦理专业培训，证书编号：LAC20220306，做过动物实验且熟练掌握本实验相关操作技能。具备完成本动物实验项目的能力。

Zhao Xinyue has received professional training in animal experimentation qualification and animal welfare ethics at the School of Life Sciences, Jilin University, Certificate No. LAC20220306, and has performed animal experiments and is proficient in the operation skills related to this experiment. She is capable of completing this animal experiment.

赵秀红，在长春维石检测技术服务有限公司接受过动物实验上岗和动物伦理培训，熟练掌握实验相关操作技能，具备完成本实验项目的能力。

Zhao Xiuhong, In Changchun Weishi Testing Technology Service Co., Ltd. she has received animal experiments and animal ethics training, and is proficient in experimental operation skills, possessing the ability to complete this experimental project.

实验动物设施许可证编号<br>Name and certificate number of the facility	SYXK（吉）2019-0007
拟实验时间：<br>Experimental period:	2024 年 2 月 1 日 至 2024 年 5 月 1 日<br>2024 Y 2 M 1 D to 2024 Y 5 M 1 D

动物实验项目的、必要性、意义和如何设计以达成研究目标的

Experimental objective, necessity and significance and how the program has been designed to achieve the objectives of the research.

实验目的：为了研究 E3 泛素连接酶 UBR5 在结直肠癌中侵袭迁移的功能及机制。

Experimental Objective: To investigate the function and mechanism of invasive migration of the E3 ubiquitin ligase UBR5 in colorectal cancer.

实验必要性：只有通过动物模型才能尽可能真实的模拟体内环境，目前尚无更好替代方法，须进行动物实验。

Experimental necessity: The best possible simulation of the in vivo environment can only be achieved through an animal model, and there is currently no better alternative to animal testing.

实验意义：本次动物实验为开发针对转移性癌症的更具针对性的创新药物提供了理论基础和潜在应用。

Experimental significance: This animal experiment provides a theoretical basis and potential application for the development of more targeted and innovative drugs for metastatic cancer.

实验设计：实验动物分五组各两只，HCT116 条件敲除空载小鼠（shNC），条件敲除 UBR5 小鼠（shUBR5）。以及条件过表达小鼠（overNC），条件过表达 UBR5 小鼠（overUBR5），条件过表达 UBR5 C2768S 小鼠（overUBR5 C2768S）。注射细胞，细胞量为 $5*10^6$，大概 3 到 4 周后，处死小鼠，剥离皮下移植瘤，每只小鼠肿瘤进行组织固定、包埋，每只小鼠进行 3 组蜡块包埋，切片（5um 厚）。

experimental design: The experimental animals were divided into five groups of two animals each, HCT116 conditional knockout null carrier mice (shNC), conditional knockout UBR5 mice (shUBR5). and conditional overexpression mice (overNC), conditional overexpression UBR5 mice (overUBR5), and conditional overexpression UBR5 C2768S mice (overUBR5 C2768S). Cells were injected with a cell volume of $5*10^6$, and after approximately 3 to 4 weeks, the mice were executed, the subcutaneous graft tumours were stripped, and the tumours of each mouse were tissue-fixed and embedded, and 3 sets of wax blocks were embedded in each mouse and sliced (5 um thick).

实验动物福利伦理审查表

<table>
<tr>
<td rowspan="3">拟使用动物信息
Animal to be used</td>
<td colspan="2">实验动物来源的生产许可证编号：北京维通利华实验动物技术有限公司 SCXK（京）2021-0006
Beijing Vital River Laboratory SCXK（京）2021-0006</td>
<td>实验动物质量合格证编号：
Certificate number
NO.110011241100999973</td>
</tr>
<tr>
<td colspan="2">品种/品系 breed/strain
☐小鼠 mice ☑裸鼠 nude mice ☐大鼠 rat
☐豚鼠 guinea pig ☐兔 rabbit
☐转基因动物 genetically modified animal
☐其他（具体说明）others</td>
<td>等级 Grade
☐普通 CV ☐清洁 CL
☑无特定病原体 SPF ☐无菌 GF
☐悉生 GN ☒其他</td>
</tr>
<tr>
<td>数量（只）Number: 10♀</td>
<td>体重 Weight 18-20g</td>
<td>周龄 Age 4-6W</td>
</tr>
<tr>
<td rowspan="3">拟开展动物实验的详细信息
Detailed information of the experiments animals on</td>
<td colspan="3">详细列出对动物可能造成的所有可预期的伤害，包括动物运输、每个实验方案动物饲养方式、实验操作步骤中等可能产生伤害或不适的细节以及拟采取防控措施，如麻醉剂名称及麻醉途径等
Description of the overall harms expected to be experienced by the animals-including details of the likely adverse effects of each protocol , cage breeding and the steps which will be taken to control these adverse effects. Such as name of anesthetic and route of anesthesia.
动物运输：实验动物的运输归供应商负责，符合国家标准。
动物饲养方式：实验动物在 IVC 屏障环境内饲养，饲养条件符合国家标准。
动物运输过程中对小鼠造成的恐慌：小鼠到达实验室后饲养一段时间后开始实验。
动物手术过程中可能造成伤口感染：专业的熟练的实验操作人员进行手术。
麻醉剂使用异氟烷吸入式麻醉。
Animal transportation: The transportation of experimental animals is responsible by the supplier, which is in accordance with national standards.
Animal rearing: Experimental animals are raised in the IVC barrier environment, and the rearing conditions are in accordance with national standards.
Fear of mice during animal transport: The mice arrive at the laboratory and are kept for a period of time before the experiment begins.
Wound infection may occur during animal surgery: A professional and skilled laboratory operator performs the surgery.
The anesthetic is an inhalation anesthetic using isoflurane.</td>
</tr>
<tr>
<td colspan="3">主要观察指标 Main observation target
小鼠肿瘤边缘、连带周围组织的切片；肿瘤体积。
Sections of mouse tumor margins, with surrounding tissue; tumor volume.</td>
</tr>
<tr>
<td colspan="3">实验动物仁慈终点或实验终结的指标、动物处死方式及尸体或残体的处理方法
Humane endpoint or experimental terminative indicator、Methods of animal death and disposal of corpses or remains
在得到实验结果或动物实验结束后，放入动物，以每分钟替换安乐死箱容积 30%-70%的速度灌注 CO2 于箱内，确定动物不动、不呼吸、瞳孔放大。关闭 CO2，再观察 2 min-3 min，确定动物死亡。
After the results of the experiment were obtained or the animal experiment was completed, the animal was placed in and CO2 was instilled in the chamber at a rate of 30%-70% of the volume of the euthanasia chamber being replaced every minute, and it was determined that the animal was immobile, not breathing, and that the pupils were dilated. Turn off the CO2 and observe for another 2 min-3 min to determine that the animal is dead.
将动物尸体放置于特定冰柜，由医疗废弃物处理公司统一处理。
The animal carcasses were placed in a specific freezer and disposed of by a medical waste disposal company.</td>
</tr>
<tr>
<td>声明
Declaration</td>
<td colspan="3">1.我将自觉遵守实验动物福利伦理相关法规和各项规定，同意接受伦理委员会和实验动物室管理者的监督与检查，如违反规定，自愿接受处罚。
2.本人保证本申请表中所填内容真实、详尽和易懂。
1. I will abide by the law and regulation stipulation, and accept the supervision and inspection by the committee and laboratory animal department , and voluntarily accept the punishment if any infringement.
2. The information I have given is accurate, detailed and comprehensive.
声明人：课题负责人签（章）：[illegible]
Declarant: Signature (stamp) of PI
动物实验负责人签(章)：[illegible]
Signature (stamp) of Director of animal experiment
2024 年 01 月 19 日
2024 Y 01 M 19 D</td>
</tr>
</table>

实验动物福利伦理审查表

审查依据 Inspectionconten ts	GB/T 35892-2018 实验动物 福利伦理审查指南<br>(Laboratory Animal - Guideline for ethical review of animal welfare)。<br>实验动物饲养管理和使用指南，第 8 版，上海科技出版社<br>(Guide for the Care and Use of Laboratory Animals: Eighth Edition) 。

申报部门意见 Opinion of applicant Department of institution

研究室负责人签（章）：赵秀红 2024 年 01 月 22 日

Signature (stamp) of the Department principal Xiuhong Zhao 2024 Y 01 M 22 D

主管医师意见 Opinion of Veterinary of institution

主管医师签（章）：单丹 2024 年 01 月 22 日

Signature (stamp) Veterinary Dan Shan 2024 Y 01 M 22 D

实验动物设施意见 Opinion from laboratory animal facility

设施负责人签（章）：孟祥鹤 Xianghe Meng 2024 年 01 月 28 日

Signature (stamp) of the facility Director 2024 Y 01 M 28 D

福利伦理委员会审批意见及审查委员表决 Approval opinion of Committee and Inspection by members:

☑同意 Agree 单丹 Dan Shan 赵秀红 Xiuhong Zhao 周旋 Xuan Zhou [illegible] Liangcang Cai [illegible] Mingquan Gong

☐不同意 Disagree

主任委员签（章）：[illegible] 2024 年 01 月 28 日

Signature (stamp) of Chairman of Committee: Liangcang Cai 2024 Y 01 M 28 D

申报说明：申报时，请提交本表一式两份及电子版。受理编号和批准文号由伦理委员会填写。

Notice: Submitting the Application Format in duplicate and a electronic edition.

The Appl. No. and IACUC Issue No. are make out by Jury.
